# Early detection of pertussis related activity by wastewater surveillance of *Bordetella pertussis-* and *Bordetella parapertussis-*associated DNA concentrations in Osaka, Japan

**DOI:** 10.1080/22221751.2026.2717860

**Published:** 2026-08-10

**Authors:** Koichiro Suzuki, Daisuke Motooka, Naomi Sakon, Kazunori Tomono

**Affiliations:** a BIKEN-RIMD NGS Laboratory, Research Institute for Microbial Diseases, The University of Osaka, Suita, Japan; b Biomedical Science Center, The Research Foundation for Microbial Diseases of Osaka University (BIKEN), Suita, Japan; c Bioinformatics Center, Research Institute for Microbial Diseases, The University of Osaka, Suita, Japan; d Department of Microbiology, Osaka Institute of Public Health, Osaka, Japan

**Keywords:** Pertussis, wastewater surveillance, *Bordetella pertussis*, *Bordetella parapertussis*, early detection

## Abstract

Pertussis causes recurrent outbreaks worldwide. Clinical surveillance can be affected by healthcare-seeking behaviour, diagnostic practices, testing sensitivity, and reporting delays. This study evaluated the utility of wastewater surveillance (WWS) by quantifying *Bordetella-*associated *IS481* and *IS1001* DNA concentrations. Weekly wastewater samples were collected from four treatment plants in Osaka Prefecture, Japan, between April 2023 and July 2025, and *IS481*- and *IS1001*-associated DNA concentrations were measured using quantitative PCR. The putative *B. pertussis*-associated *IS481* DNA concentration increased markedly from 2024 onwards. Both threshold-based onset detection and cross-correlation analysis indicated that the wastewater *IS481* DNA concentration preceded reported cases by one to several months. In contrast, the *B. parapertussis-associated IS1001* DNA concentration exhibited a transient peak in 2023, which aligned with contemporaneous clinical reports but remained at low levels thereafter. These findings suggest that WWS may detect increases in pertussis-related activity earlier than clinical reporting and highlight the potential of WWS as a complementary tool to clinical surveillance for monitoring respiratory infectious diseases.

## Introduction

Accurate estimation of the number of infected individuals is essential for controlling the spread of infectious diseases. However, surveillance based solely on clinical diagnoses often fails to provide sufficient information for public health interventions because it is affected by disparities in diagnostic capacity, healthcare access, symptom presentation, and healthcare-seeking behaviour. Because respiratory infections spread more easily and cause repeated outbreaks more frequently than diarrhoeal, skin, or sexually transmitted infections, rapid and region-specific monitoring methods are required.

Respiratory pathogens include viruses, such as influenza and SARS-CoV-2, and bacteria, including *Streptococcus pneumoniae*, *Mycoplasma pneumoniae*, and *B. pertussis*. Pertussis is caused by *B. pertussis* and can be prevented by vaccination. Although vaccination has substantially reduced the overall incidence, periodic resurgences continue to arise due to waning immunity in affected individuals, pathogenic adaptation, and underdiagnosis. A WHO-supported modelling study estimated 24.1 million pertussis cases globally in 2014, with approximately 160,700 deaths among children aged <5 years [1]. The actual burden is likely higher than the reported burden, with underreporting and misdiagnosis contributing to the lower reported burden, particularly in low-resource settings. Between 2023 and 2024, outbreaks were reported in regions with high vaccination rates, including Europe, the United States, China, and South Korea [2–5]. An outbreak was also reported in Japan in 2025 [6]. In Japan, pertussis prevention relies on a combined diphtheria, tetanus, acellular pertussis, inactivated poliovirus, and *Haemophilus influenzae* type b vaccine. Three primary doses are administered between 2 and 12 months of age, followed by a booster before 7 years and 6 months. The coverage of the primary series exceeds 96% [7]. However, booster vaccination during school age and adolescence has not been implemented, resulting in low booster coverage among older children and adults. Consequently, only approximately 50% of individuals aged 8 years or older have antibodies against pertussis toxin [8]. In contrast, the United States recommends booster immunization with tetanus, diphtheria, and acellular pertussis vaccine at 11–12 years, which has been shown to reduce the risk of infection due to waning immunity and prevent secondary transmission to infants [9,10]. In Japan, a higher prevalence of antibodies among adolescents and adults has been reported than that among younger children [8], likely reflecting natural infection. This raises concerns regarding the likelihood of older children and adults, many of whom were previously vaccinated but may have waning immunity, serving as household sources of infection for unvaccinated infants [11–13]. Infants <1 year of age are particularly vulnerable because they are unvaccinated or incompletely vaccinated. Therefore, family booster vaccinations and strict infection prevention measures are critical. In Europe, approximately 70% of pertussis-related deaths occur in infants <6 months of age, and the case-fatality rate in this age group is estimated at 1–2% [14]. In Japan, four infant deaths were reported in 2025 [15]. Given the high mortality risk, enhanced protection of infants remains a public health priority. However, pertussis is often underdiagnosed during interepidemic periods, particularly in adolescents and adults who frequently lack classic symptoms. Early catarrhal-stage symptoms are nonspecific and mild, and during the paroxysmal stage, culture sensitivity declines, making PCR or culture confirmation challenging [16]. Consequently, clinical reporting alone cannot provide timely and accurate assessments of epidemic trends.

Monitoring *B. parapertussis* is also important for accurately assessing the true burden of pertussis-like illness. This close relative of *B. pertussis* causes pertussis-like disease in humans. Transmission occurs via aerosols, and symptoms resemble those of pertussis but are generally milder and shorter in duration. Globally, *B. parapertussis* accounts for 2–20% of pertussis-like cases [17]. In Japan, an epidemiological study conducted at a pediatric medical centre in Tokyo detected *B. parapertussis* DNA in ∼1.0% of respiratory samples in 2023 [18]. In the United States, reemergence of *B. parapertussis* was reported from 2019 to 2023, underscoring its regional epidemic potential and public health relevance [19]. Therefore, reliance on clinical diagnoses and case-based reporting carries a risk of underestimating the true burden of pertussis and related illnesses, particularly among adolescents and adults with mild or asymptomatic infection. This also complicates the assessment of the risk of infant transmission. Thus, integrated surveillance targeting both *B. pertussis* and *B. parapertussis* is required.

Wastewater surveillance (WWS) has recently gained attention as a valuable complement to clinical surveillance for early detection and monitoring of infectious diseases at the population level. Detecting pathogen-derived nucleic acids in wastewater is noninvasive, cost-effective, and independent of healthcare-seeking behaviour or diagnostic testing, enabling the capture of symptomatic and asymptomatic infections in near real time. For respiratory pathogens, nucleic acid signals in wastewater may arise from multiple routes, including swallowed respiratory secretions, oral or nasal secretions, sputum, and domestic wastewater from hygiene-related activities. Importantly, the primary advantage of WWS lies in its ability to capture infection dynamics at the population level rather than at the level of individual shedding duration, thereby enabling assessment of the timing and progression of community-wide transmission over extended periods. This approach has been applied to poliovirus, SARS-CoV-2, and influenza [20–22] and may detect increases in community-level pathogen activity earlier than clinical reporting [23–25]. WWS has also been used for enteric bacterial pathogens and antimicrobial-resistant organisms [26,27]. Moreover, aircraft wastewater monitoring is effective for tracking the international spread of antimicrobial resistance [28]. Thus, WWS can be applied across a broad spectrum of pathogens, providing objective epidemic indicators that supplement clinical surveillance by capturing early epidemic signals that would otherwise be missed.

This study aimed to evaluate whether WWS can be used to monitor pertussis-related activity by measuring *Bordetella*-associated *IS481* and *IS1001* DNA concentrations in wastewater.

## Materials and methods

### Wastewater sample collection and nucleic acid extraction

The study was conducted in Osaka Prefecture, Japan, the third most populous prefecture in the country, with a population of 8.8 million and the second highest population density after Tokyo (4,600 persons/km²). Wastewater samples were collected from four treatment plants (Sites A-D) in Osaka Prefecture that collectively serve approximately 1.4 million people (approximately 16% of the total population of the prefecture). Sampling was conducted weekly from April 2023 to July 2025. At each facility, 1 L of influent wastewater was obtained by grab sampling. Samples were transported at 4 °C and processed within 24 h after collection. Before aliquoting, each 1 L wastewater sample was thoroughly mixed to reduce subsampling variability, and a 40 mL aliquot was used for nucleic acid extraction. The aliquot was centrifuged at 12,000 × g for 30 min, and the resulting pellet fraction, which may contain bacterial cells, cell debris, particle-associated DNA, and suspended solids, was resuspended in 600 µL of influent wastewater. DNA and RNA were extracted using the InnuPure C16 touch system with the InnuPREP Virus DNA/RNA Kit-IPC16 (Analytik Jena, Jena, Germany).

### Measurement of IS481- and IS1001-associated Bordetella DNA concentrations using multiplex quantitative PCR

The extracted nucleic acids were analyzed using multiplex qPCR targeting the insertion sequences *IS481* and *IS1001*. *IS481* is a high-copy insertion sequence known to provide high sensitivity for *B. pertussis* detection [29], whereas *IS1001* is commonly used for the specific detection of *B. parapertussis* [30]. The primer and probe sequences used in this study are provided in Supplementary Table 1. Quantification was performed using the Biomark HD system (Standard BioTools, San Francisco, CA, USA) with the 192.24 Dynamic Array IFC plate, Preamp Master Mix, and TaqMan Fast Advanced Master Mix (all from Standard BioTools, San Francisco, CA, USA). Because *Bordetella*-associated target DNA was expected to be present at low concentrations in wastewater, qPCR was performed after a preamplification step using 5 μL of extracted nucleic acid, following the standard protocol of the Biomark HD system. To assess analytical performance, we compared the single-copy gene prn and the multicopy insertion sequence *IS481* and evaluated assay performance with and without preamplification (Figure S1(A and B)). Potential wastewater matrix effects were evaluated separately using *B. pertussis*-spiked wastewater samples, as described below (Figure S1(C)). Based on these comparative evaluations, *IS481* was selected as the primary target for wastewater analysis. Each sample was analyzed in duplicate, with nuclease-free water as the negative control. Samples were considered positive when Ct values were ≤ 29, based on experimentally determined operational detection limits.

### Evaluation of wastewater matrix effects using B. pertussis-spiked samples

To evaluate potential inhibitory effects of the wastewater matrix on *IS481* detection, a spike-in experiment was performed using cultured *B. pertussis* strain 18323. The strain was cultured on Bordet-Gengou agar supplemented with 1% glycerol, 20% defibrinated horse blood, and ceftibuten, and subsequently suspended in Stainer-Scholte medium. Colony-forming units (CFU) were estimated from optical density at 650 nm using the equation 1.0 OD_650_ = 3.0 × 10⁹ CFU/mL. Serial dilutions of cultured *B. pertussis* were spiked into wastewater samples and processed using the same workflow as the study samples. Briefly, nucleic acid extraction was performed using the InnuPure C16 touch system with the InnuPREP Virus DNA/RNA Kit-IPC16 (Analytik Jena, Jena, Germany). Extracted nucleic acids were subjected to preamplification using KOD plus ver.2 DNA polymerase (KOD-211; TOYOBO, Osaka, Japan) on an Applied Biosystems™ ProFlex™ PCR System (Thermo Fisher Scientific, Waltham, MA, USA). The preamplified products were subsequently purified using AMPure XP beads (Beckman Coulter, Brea, CA, USA). Quantitative PCR targeting *IS481* was then performed using One Step PrimeScript™ III RT-qPCR Mix, with UNG (RR601A; Takara Bio, Shiga, Japan) on a QuantStudio® 5 Real-Time PCR System (Applied Biosystems, Thermo Fisher Scientific, Waltham, MA, USA). This experiment was used to assess whether wastewater matrix-derived inhibition substantially affected detection of the *IS481* DNA concentration.

### Data analysis

*IS481*- and *IS1001* DNA concentrations were converted to target-copy estimates per litre of wastewater for temporal comparison. These estimates were used as semi-quantitative indicators of wastewater DNA concentration and were not interpreted as bacterial cell numbers, genome equivalents, or infection counts. The operational detection threshold was defined as Ct ≤ 29 based on experimentally determined assay performance, and values meeting this criterion were used for time-series analysis. The mean value across the four treatment facilities was calculated as the arithmetic mean, since there were no substantial differences in the population served by each facility. During the entire study period, only one wastewater measurement was missing owing to the New Year holiday period. This single missing value was imputed using linear interpolation to maintain continuity of the time series for moving-average and cross-correlation analyses. The averaged values were used for subsequent analyses. Aggregating data across sites has been reported to improve the ethical handling and statistical robustness of wastewater-based epidemiology [31]. Based on previous clinical surveillance results indicating that pertussis epidemics typically persist for more than six months (Figure S2), concentration data were plotted as 4-week moving averages to minimize short-term fluctuations caused by environmental and sampling variability and to capture overall outbreak trends more accurately [32].

### Threshold-based detection of increases in wastewater DNA concentration and reported cases

The pre-increase low-activity period was defined as the baseline to identify the onset points of sustained increases in each time series. For reported pertussis cases, weekly case counts from January 2022 to December 2023 were used as the low-activity baseline period, during which case counts remained low. For the wastewater *IS481* DNA concentration, October to December 2023 was used as a fixed pre-epidemic baseline period, during which the *IS481* DNA concentration remained consistently low before the sustained increase. The baseline mean (μ) and standard deviation (σ) were calculated, and a threshold was set at μ + 2σ to identify signals exceeding background variability. The first week of at least four consecutive weeks in which the series exceeded this threshold was defined as the onset of a sustained increase. The difference between the case-based and wastewater-signal-based onset weeks (cases – wastewater) was calculated, with positive values indicating that the wastewater signal increased earlier than the case reports. Sensitivity analyses varied the threshold among the baseline mean + 1.5σ, +2.0σ, and +2.5σ and the consecutive-week requirement from two to five weeks. When one parameter was varied, the other was held at its primary value (the baseline mean + 2σ or four consecutive weeks). The wastewater onset week, case onset week, and onset difference were calculated for each specification. Similar threshold-based approaches have been widely applied in outbreak detection because of their interpretability and robustness [33,34]. This threshold-based analysis was used as an exploratory method to describe temporal relationships and was not intended to establish a validated outbreak prediction model.

### Cross-correlation analysis

To evaluate the temporal lead-lag relationships between *IS481* DNA concentrations and case counts, cross-correlation functions (CCFs) were calculated over lags from −20 to +20 weeks. For each lag (ℓ), overlapping segments of the two time series were z-standardized, and the Pearson correlation coefficient r(ℓ) was computed. Positive lags (ℓ > 0) indicated that DNA trends led to reported cases by ℓ weeks, whereas negative lags indicated the reverse. The lag with the highest positive correlation was reported. To evaluate whether the estimated lead-lag relationship depended on the smoothing window, CCF analyses were performed using 2-week, 3-week, and 4-week moving averages. CCF analysis is a widely used method for quantifying temporal precedence in environmental pathogen surveillance [35,36]. In this study, CCF analysis was used to describe temporal association and was not interpreted as evidence of causality or validated forecasting performance.

### Computational analysis

All statistical analyses, including outbreak onset detection and cross-correlation analysis, were implemented using custom Python scripts available at https://bitbucket.org/daisukem/pertussis_wws.

## Results

### Analytical evaluation of IS481 detection in wastewater

In wastewater samples spiked with serial dilutions of cultured *B. pertussis* strain 18323, the *IS481*-associated signal increased in a dose-dependent manner after nucleic acid extraction, preamplification, and qPCR, suggesting that substantial wastewater matrix-derived inhibition was not apparent under the conditions used in this study (Figure S1).

### Temporal dynamics of the B. pertussis-associated IS481 DNA concentration in wastewater

**Figure 1.**
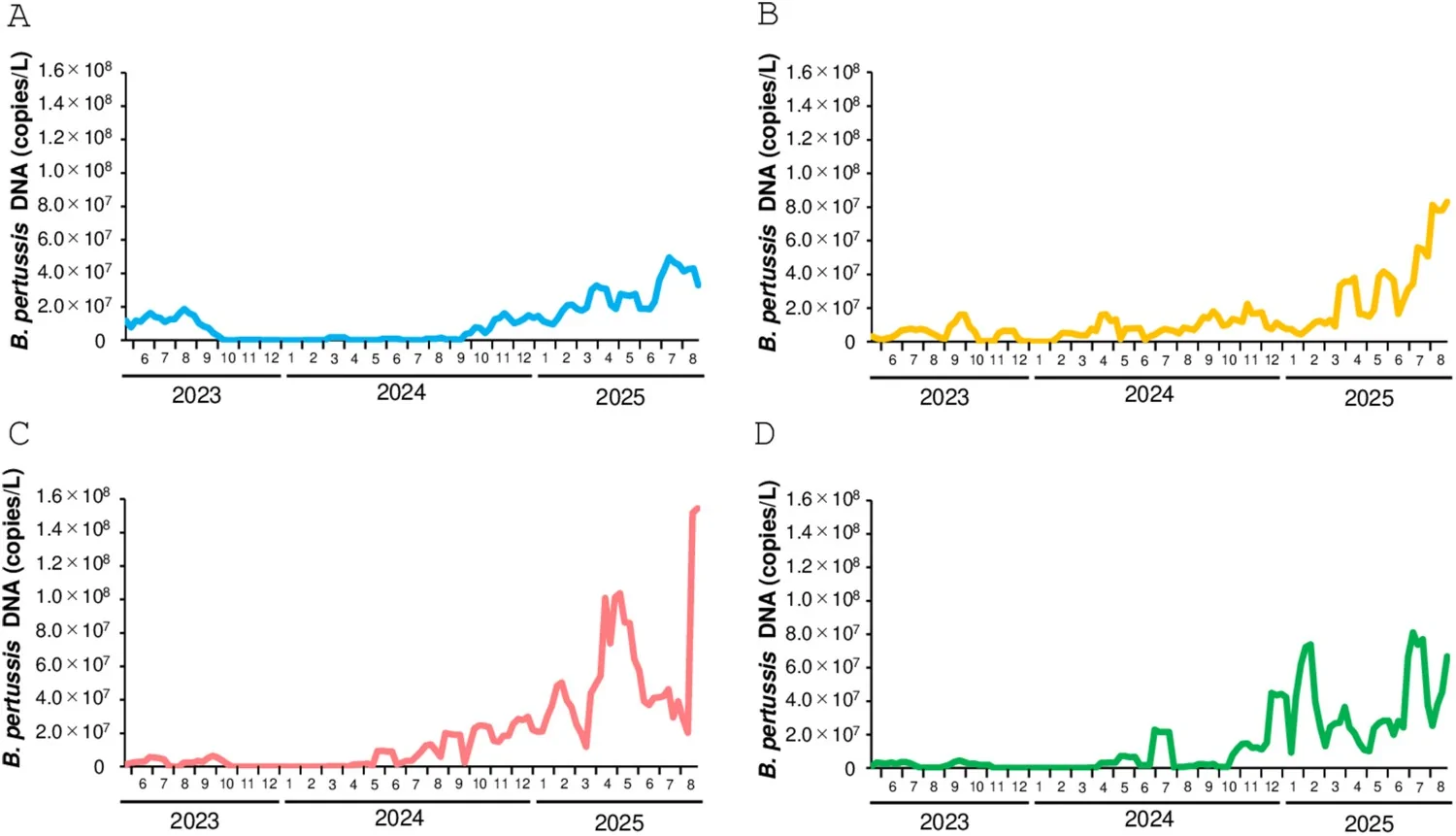
Detection of *B. pertussis*-associated *IS481* DNA in wastewater from four sites in Osaka, Japan. Four-week moving averages of *B. pertussis-associated IS481* DNA concentrations are shown for wastewater samples collected from four sites in Osaka Prefecture (A, Site A; B, Site B; C, Site C; and D, Site D). DNA concentrations varied by site and increased during periods corresponding to elevated pertussis case reports in the region. These data indicate that wastewater surveillance can capture the localized fluctuations in pathogen circulation.

At all four treatment plants, the influent wastewater samples exhibited a gradual increase in the putative *B. pertussis*-associated *IS481* DNA concentration beginning in mid-2024 (Figure 1). At Site A, the *IS481* DNA concentration rose sharply around epidemiological week 38 of 2024, reaching a maximum target-copy estimate of 4.9 × 10^7^ copies/L in week 28 of 2025. At Site B, the *IS481* DNA concentration began increasing around week 7 of 2024, peaking at 8.1 × 10^7^ copies/L in week 31 of 2025. At Site C, increases were observed from approximately week 34 of 2024, with a peak of 1.0 × 10^8^ copies/L in week 18 of 2025. At Site D, the *IS481* DNA concentration steadily rose from around week 43 of 2024, reaching a maximum of 8.1 × 10^7^ copies/L in week 27 of 2025.

### Temporal relationship between the wastewater IS481 DNA concentration and reported cases

**Figure 2.**
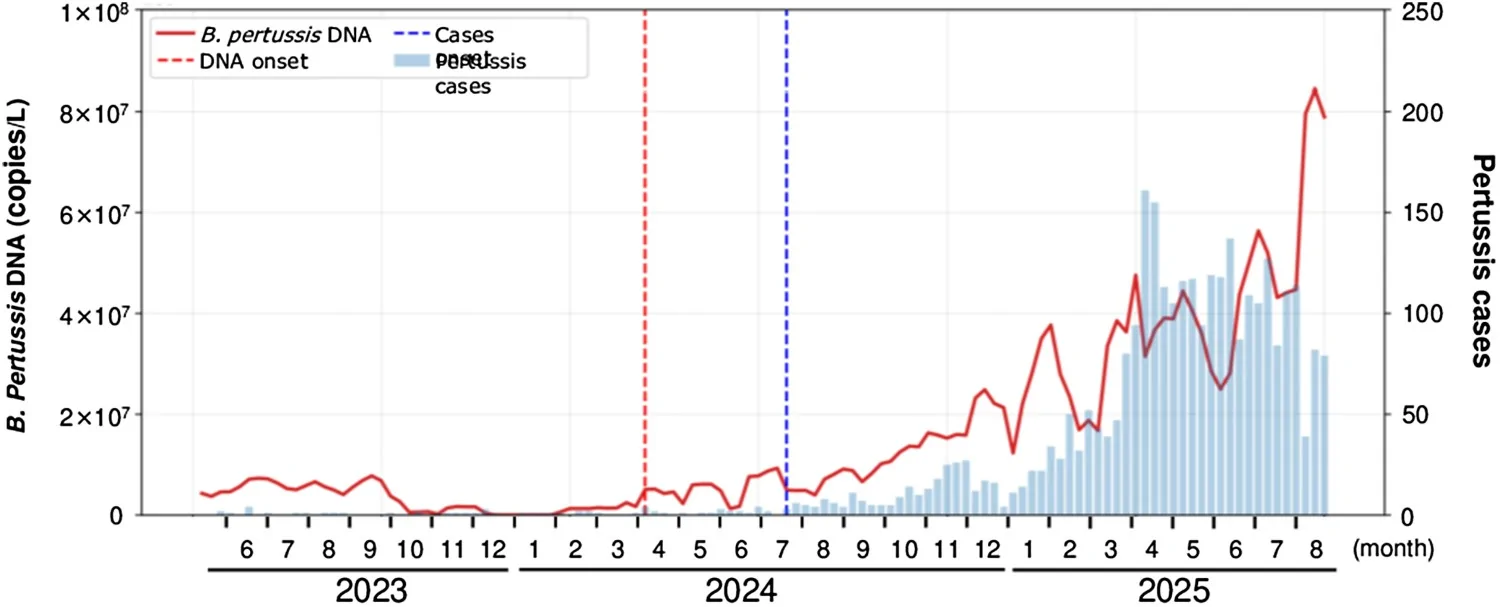
Temporal trends of *B. pertussis*-associated *IS481* DNA in wastewater and pertussis cases. Weekly concentrations of *B. pertussis*-associated *IS481* DNA in wastewater (red line, left y-axis) and reported pertussis cases (light blue bars, right y-axis) in Osaka, Japan. Dashed vertical lines indicate the onset of increases in DNA concentration (red) and case reports (blue), under the primary analytical definition, defined as the first week exceeding the baseline mean + 2SD for at least four consecutive weeks. Sensitivity analyses using alternative threshold values and consecutive-week requirements are presented in Supplementary Table S3. Under the primary analytical definition, the increase in wastewater *IS481* DNA concentration preceded the increase in reported cases.

Weekly time-series data of *B. pertussis*-associated *IS481* concentration, calculated as 4-week moving averages and averaged across the four sites, were compared with the reported pertussis case counts in Osaka Prefecture, obtained from the Osaka Infectious Disease Surveillance Center [32]. Both the wastewater *IS481* DNA concentration and reported pertussis case counts exhibited upward trends from mid-2024–2025, although the timing of their onset differed (Figure 2). To describe this temporal relationship, threshold-based onset detection and cross-correlation analyses were conducted.

For threshold-based onset detection, the pre-increase low-activity period was defined as the baseline. The threshold was set as the baseline mean plus two standard deviations (SD), and the onset week was defined as the first week of at least four consecutive weeks in which the value exceeded this threshold. Using this criterion, the onset of the increase in wastewater *IS481* DNA concentration was identified in week 15 of 2024 (April 8–14), whereas the onset of the case increase was detected in week 30 of 2024 (July 22–28), resulting in a difference of +15 weeks (cases – wastewater) (Figure 2, Table 1). The estimates ranged from 15 to 16 weeks when the threshold was varied and from 4 to 15 weeks when the consecutive-week requirement was varied. Across all analytical specifications, the wastewater IS481 DNA concentration consistently preceded reported cases, with overall onset differences ranging from 4 to 16 weeks (Supplementary Table S3). Cross-correlation analysis was conducted using z-standardized time-series data over lags of −20 to +20 weeks. In the main analysis using 4-week moving averages, the maximum positive correlation was observed at a lag of +10 weeks (Table 1). Sensitivity analyses using 2-week and 3-week moving averages also identified a positive 10-week lag, indicating that the temporal relationship between the wastewater *IS481* signal and reported cases was not dependent on a single moving-average window. Threshold-based onset detection and cross-correlation analysis assess different aspects of the temporal relationship and should not be interpreted as estimating the same quantity. These findings suggest that, in this dataset, the wastewater *IS481* DNA concentration preceded the increase in reported cases on the order of one to several months; however, these analyses were exploratory and did not establish forecasting performance.

**Table 1.** Summary of onset detection and cross-correlation analysis between *B. pertussis-*associated *IS481* DNA concentrations in wastewater and pertussis case counts.

Item	Value
Wastewater onset week (week_number)	8–14 April 2024 (2024 week 15)
Case onset week (week_number)	22–28 July 2024 (2024 week 30)
Onset difference (cases – wastewater) [weeks]	15
Maximum correlation lag (+=DNA leads)	10
Maximum correlation coefficient (positive correlation)	0.883
*IS481* DNA threshold (mean + 2SD, baseline)	2,665,542
Case threshold (mean + 2SD, baseline)	2.57
Baseline mean reported cases (2022–2023)	0.76
Study period used for onset and cross-correlation function (CCF) analyses	2024–2025
Consecutive exceedance weeks	4

SD, standard deviation.

This table summarizes onset detection results and cross-correlation analysis between putative *B. pertussis*-associated *IS481* DNA concentrations in wastewater and reported pertussis cases. Onset was defined as the first week in which the values exceeded the baseline mean by two standard deviations for four consecutive weeks. The difference in onset timing (cases minus DNA) indicates whether the wastewater signals preceded or followed the increase in clinical cases. Cross-correlation functions (CCFs) were computed over lags of ±20 weeks, with positive lags representing DNA-leading cases. The table presents the onset weeks, onset difference, maximum correlation lag and coefficient. The threshold-based onset results shown in this table were obtained using the primary analytical definition of the baseline mean + 2SD and four consecutive weeks above the threshold. Sensitivity analyses using alternative threshold values and consecutive-week requirements are presented in Supplementary Table S3.

### Detection of B. parapertussis-associated IS1001 DNA concentration in wastewater

**Figure 3.**
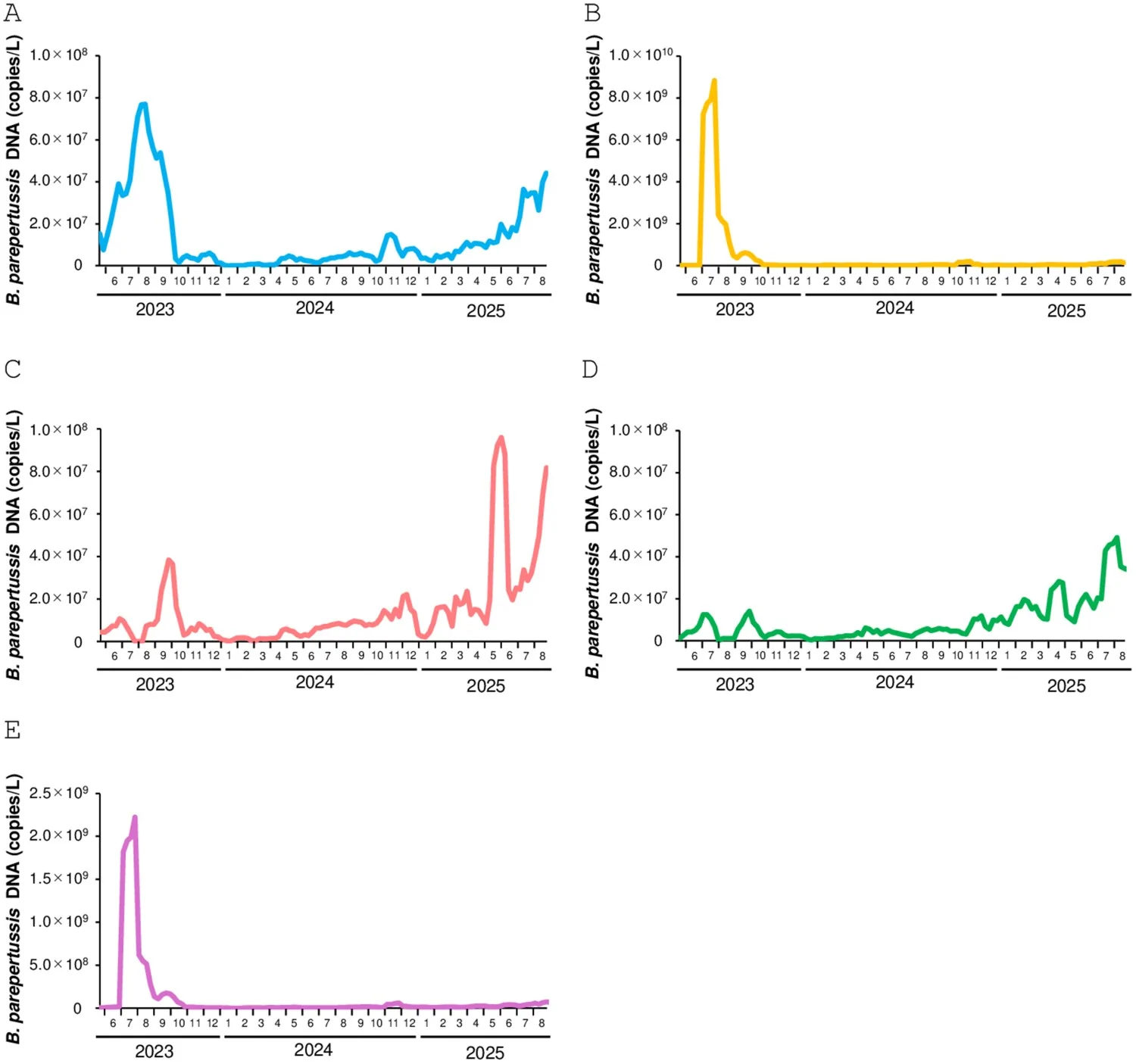
Detection of *B. parapertussis*-associated *IS1001* DNA in wastewater from four sites in Osaka, Japan. Four-week moving averages of *B. parapertussis*-associated *IS1001* DNA concentrations are shown for wastewater samples collected from four sites in Osaka Prefecture (A, Site A; B, Site B; C, Site C; and D, Site D). Panel E shows the average across all four sites. Concentration levels were generally lower and more variable than those of *B. pertussis;* however, site-specific increases were observed. These findings suggest that wastewater surveillance can simultaneously track the circulation of both *B. pertussis* and *B. parapertussis* at the community level.

The *B. parapertussis*-associated *IS1001* DNA concentration was also calculated as 4-week moving averages. At Site A, the *IS1001* DNA concentration peaked at 7.7 × 10^7^ copies/L in week 32 of 2023. At Site B, a peak of 8.8 × 10^9^ copies/L was observed in week 29 of 2023. At Site C, the maximum concentration of 9.5 × 10^7^ copies/L occurred in week 22 of 2025, while at Site D, concentrations reached 4.9 × 10^7^ copies/L in week 29 of 2025, although overall levels remained low. Averaged across all sites, a transient peak was observed in week 29 of 2023, followed by relatively low levels after 2024 (Figure 3).

## Discussion

In this study, we evaluated whether wastewater surveillance could capture pertussis-related activity by measuring *Bordetella*-associated *IS481*- and *IS1001* DNA concentrations in wastewater influent. Increases in putative *B. pertussis*-associated *IS481* DNA concentration in wastewater were observed substantially earlier than increases in reported cases of pertussis. Under the primary threshold-based definition, wastewater onset preceded case onset by 15 weeks, while sensitivity analyses yielded onset differences ranging from 4 to 16 weeks. Cross-correlation analysis consistently identified a 10-week wastewater-leading lag across 2-week, 3-week, and 4-week moving averages. Although these analyses assessed different aspects of the temporal relationship, both indicated that the wastewater *IS481* DNA concentration preceded the reported cases by one to several months. The high correlation coefficient (0.883) obtained from this cross-correlation analysis indicates that, after correcting for temporal shifts, the fluctuation pattern of *B. pertussis*-associated *IS481* DNA concentrations in wastewater closely resembled the trend in reported case counts (Table 1). This temporal association supports the potential of wastewater surveillance as a complementary population-level indicator of pertussis-related activity, although the present study did not establish a forecasting model or validate predictive performance. These temporal differences should not be interpreted as the incubation period of pertussis or as fixed biological intervals between infection and clinical disease. Rather, they represent population-level temporal differences between wastewater signals and reported cases under the analytical conditions used in this study. Clinical surveillance is influenced by healthcare-seeking behaviour, diagnostic practices, testing sensitivity, physician awareness, and reporting processes, whereas wastewater surveillance may capture aggregated signals from symptomatic, mildly symptomatic, and asymptomatic individuals within the catchment population. Nevertheless, the estimated lead time may depend on the moving-average window, baseline definition, threshold setting, and consecutive-week criterion, and should therefore be interpreted cautiously. The onset detection method used in this study statistically defined deviations from the baseline and automatically identified the point at which an increase began. Its effectiveness has also been validated for trend analysis of SARS-CoV-2 RNA in wastewater [34]. In addition, cross-correlation analysis is widely used in the field of wastewater epidemiology as a method for quantifying the temporal relationship between pathogen concentration and the number of clinical cases, with its robustness demonstrated in multiple studies focusing on COVID-19 [36]. However, these analyses should be regarded as exploratory approaches for describing temporal association, rather than as evidence of definitive predictive performance.

The temporal difference observed between the wastewater *IS481* DNA concentration and reported cases may reflect pertussis-related activity that was not fully captured by clinical surveillance, including underdiagnosed, asymptomatic, or mildly symptomatic cases, because bacterial shedding continues even in the absence of clinical reporting. In Japan, clinical surveillance for pertussis was revised in 2018 to improve the case ascertainment. Based on the National Epidemiological Surveillance of Infectious Diseases system, weekly reporting of clinically diagnosed pertussis transitioned from approximately 3,000 pediatric sentinel sites to a full enumeration [37]. However, clinical surveillance remains influenced by healthcare-seeking behaviour, testing practices, diagnostic sensitivity, and physician awareness. The temporal difference observed in this study may reflect underdiagnosed, mildly symptomatic, asymptomatic, or unreported infections, as well as delays related to healthcare-seeking, testing, diagnosis, and reporting. Gill et al. reported that asymptomatic infections in adults and vaccinated individuals may contribute to sustaining pertussis transmission [38], and our findings are consistent with this possibility. Moreover, reduced culture sensitivity and atypical clinical presentations, which are known to contribute to reporting delays [39], may also explain the observed temporal lead.

Wastewater-based pertussis surveillance reports are limited. Kim et al. conducted 1-year wastewater monitoring in Yongin, South Korea, targeting 15 respiratory viruses and 7 bacterial species using PCR. *B. pertussis* was detected at a low frequency (1%, 2 of 144 samples), confirming its presence despite limited detection [40]. However, quantitative time-series analysis and temporal comparison with regional clinical surveillance have not been fully explored. Similarly, Fu et al. reported that in Xi’an and Nanchang, *B. pertussis* DNA in wastewater preceded clinical reports by approximately 10 days [41]. Although these studies indicate the potential for early detection, the consistency and generalizability of temporal relationships between wastewater measurements and clinical reports remain unclear. In contrast, the temporal difference observed in the present study was longer than the approximately 10-day interval reported by Fu et al. This discrepancy may have been influenced by both epidemiological factors, such as the size of the target population, the regional status of pertussis outbreaks, vaccination history, and the establishment of reporting systems, as well as technical factors, including detection methods, sampling frequency, and the scale of sewage treatment plants. Previous studies have reported that the actual number of diagnosed and reported pertussis cases is often underestimated, particularly among adolescents and adults [42]. The sensitivity of *B. pertussis* detection in clinical settings may be lower during the early stages of an outbreak. Therefore, the earlier increase in wastewater *IS481*-associated DNA concentration observed in this study may reflect a combination of clinical under-ascertainment, delayed reporting, and differences in wastewater surveillance design. The present findings suggest that wastewater *IS481* DNA concentration may capture changes in pertussis-related activity before these changes become apparent in clinical reports, although further validation is required to determine the consistency and generalizability of this temporal relationship.

The temporal pattern of the *B. parapertussis*-associated *IS1001* DNA concentration differed from that of the putative *B. pertussis*-associated *IS481* DNA concentration, with the *IS1001* signal showing a transient peak in week 29 of 2023 (Figure 3). Okamoto et al. reported outbreaks in pediatric populations in Tokyo [18], and the temporal pattern observed in wastewater may be consistent with regional circulation of *B. parapertussis*-related DNA, although direct clinical data from the wastewater catchments were not available. From 2024 onward, the *B. parapertussis*-associated *IS1001* DNA concentration remained relatively low, suggesting that sustained high-level *IS1001* signal was not observed during this period. However, *IS481* and *IS1001* are multicopy insertion sequences with different copy numbers, assay characteristics, and species specificities; therefore, their absolute values should not be directly compared as measures of bacterial abundance, genome copies, or infection burden. Accordingly, the *IS1001* results should be interpreted as temporal changes in a *B. parapertussis*-associated wastewater DNA concentration rather than as direct estimates of *B. parapertussis* infection burden.

A transient increase in the *IS481* DNA concentration was observed in summer 2023 without a corresponding increase in reported cases. The reason for this discrepancy is unclear and may include unreported or mild infections, changes in healthcare-seeking or testing behaviour, environmental variability, or contributions from non-*B. pertussis IS481*-positive *Bordetella* species. Changes in population movement and infection-prevention behaviour after the COVID-19 pandemic may also have influenced respiratory infection dynamics: the number of foreign visitors to Japan recovered to approximately 25 million in 2023, about 80% of the pre-pandemic level in 2019 [43], whereas a substantial proportion of the Japanese population continued personal infection-prevention behaviours, including mask-wearing, hand hygiene, and ventilation, even after the downgrading of COVID-19 in May 2023 [44]. However, these factors should be regarded as background context, and the present study cannot establish their causal relationship with the transient increase in wastewater *IS481* DNA concentration.

In Japan, sentinel surveillance for acute respiratory infections (ARIs), including pertussis, was initiated in April 2025 to monitor disease trends and rapidly detect emerging or re-emerging infections. However, infections in asymptomatic patients or those with mild disease who do not seek clinical care may be underrepresented in clinical surveillance, whereas WWS may provide complementary population-level information. Integrating wastewater monitoring with ARI sentinel surveillance could support earlier recognition of changes in regional pertussis-related activity and help guide decisions on enhanced clinical awareness, testing, and reporting. If further validated, earlier recognition of increases in wastewater *IS481* DNA concentration could support public health preparedness, including risk communication, reinforcement of infection-prevention measures, and preparation of healthcare resources.

This study has some limitations. First, it was restricted to four wastewater treatment areas within Osaka Prefecture, and generalization to other regions should be undertaken with caution. The four wastewater treatment plants represented large urban catchments, and the results may not be directly applicable to regions with lower population density, smaller catchments, different sewer infrastructure, or different clinical surveillance systems. In addition, wastewater data were obtained from specific treatment plant catchments, whereas reported pertussis cases were aggregated at the prefectural level; therefore, a geographic mismatch between wastewater and clinical surveillance data should be considered. Second, wastewater DNA concentrations may be influenced by population size and characteristics, meteorological conditions, and wastewater treatment processes; additional analyses with adjustments for these factors are warranted. Additionally, the observed lead time between wastewater *IS481* DNA concentrations and reported clinical cases should be interpreted as a hypothesis-generating observation, rather than as evidence of a causal or empirical relationship. Because wastewater surveillance captures aggregated signals from symptomatic and asymptomatic individuals at the community level, increases in wastewater DNA concentrations may precede the reported clinical incidence. Further studies are required to establish an empirical relationship between the dynamics of *B. pertussis*-associated *IS481* DNA in wastewater and subsequent clinical incidence. Third, qPCR-based detection does not directly assess bacterial infectivity or virulence. In particular, *IS481*, which is widely used for detecting *B. pertussis*, has a high copy number and is highly sensitive. However, the copy number of *IS481* varies among strains [29], which limits the accurate estimation of bacterial cell numbers. Additionally, because *IS481* is also present in organisms, such as *B. holmesii*, its species specificity is limited when used as a single target. However, a recent U.S. study that re-tested IS481- or pIS1001-positive clinical specimens from commercial laboratories using CDC species-specific assays reported that B. holmesii accounted for only 1.4% of specimens and concluded that commercial PCR assays are reliable diagnostic tools for most patients with suspected pertussis [45]. Single-copy genes, such as *ptxA* and *prn*, offer higher specificity and would ideally serve as primary targets. However, their analytical sensitivity may be insufficient for wastewater samples. As shown in Supplementary Table S2, reliable amplification of the single-copy gene prn required ≥100 genomic DNA copies, regardless of whether preamplification was applied, indicating that low concentrations during the early phase of an outbreak may not be detected using single-copy targets. In contrast, the multicopy *IS481* assay produced detectable signals at substantially lower template inputs. As shown in Supplemental Figure S1(A and B), standard curves generated with and without preamplification showed no substantial systematic bias, supporting the analytical validity of the preamplification approach. In addition, experiments using wastewater samples spiked with cultured *B. pertussis* demonstrated that the wastewater matrix did not introduce substantial inhibition of the assay (Figure S1(C)). In wastewater surveillance, high analytical sensitivity is essential because pathogen DNA is often highly diluted in environmental samples. Although *IS481*-based quantification does not permit precise estimation of bacterial cell numbers, targeting multicopy sequences such as *IS481* enables reliable detection of low-level signals and supports the identification of early changes in community transmission dynamics. Future studies should include assessments of bacterial culturability, development of quantitative risk models, and comparative analyses across regions. Despite these limitations, this study demonstrates the potential of wastewater epidemiology, which warrants validation in large-scale, multi-region studies.

## Conclusion

This study provides a community-level wastewater-based epidemiological analysis of *Bordetella*-associated *IS481*- and *IS1001* DNA concentrations in Osaka, Japan. We observed that the putative *B. pertussis*-associated *IS481* DNA concentration in wastewater increased several weeks to several months before the increase in reported pertussis cases in this dataset. This temporal relationship may reflect clinical underascertainment, mildly symptomatic or asymptomatic infections, healthcare-seeking behaviour, testing practices, reporting delays, and differences between wastewater catchments and clinical surveillance areas. In this context, WWS may provide a complementary population-level approach for capturing temporal trends in pertussis-related activity that are not fully represented by clinical surveillance. Concurrent monitoring of *IS481*- and *IS1001*-associated DNA concentrations may also help describe temporal patterns of *B. pertussis*- and *B. parapertussis*-related activity, although these targets should not be interpreted as direct measures of bacterial burden or infection counts. These findings suggest that wastewater-based monitoring can supplement conventional clinical surveillance for earlier recognition of changes in community-level pertussis-related activity. Future applications across multiple regions and time periods, together with systematic evaluation of lead-time consistency and influencing factors (e.g. sampling frequency, meteorological conditions, and healthcare-seeking behaviour), will be essential for validating this surveillance approach and defining its role in public health practice.

## Supplementary Material

Supplemental Material

Supplemental Material

FigS2.tiff

## References

[1] Yeung KHT, Duclos P, Nelson EAS, et al. An update of the global burden of pertussis in children younger than 5 years: a modelling study. Lancet Infect Dis. 2017;17:974–980. doi:10.1016/S1473-3099(17)30390-0 28623146

[2] Bricks LF, Vargas-Zambrano JC, Macina D. Epidemiology of pertussis after the COVID-19 pandemic: analysis of the factors involved in the resurgence of the disease in high-, middle-, and low-income countries. Vaccines (Basel). 2024;12:1346. doi:10.3390/vaccines12121346 39772008 PMC11679829

[3] Christie CDC. Resurgence of pertussis: whopping the ‘100-day cough’. Curr Opin Pediatr. 2025;37:508–516. doi:10.1097/MOP.0000000000001486 40842393 PMC12422605

[4] Sheng Y, Ma S, Zhou Q, et al. Pertussis resurgence: epidemiological trends, pathogenic mechanisms, and preventive strategies. Front Immunol. 2025;16:1618883. doi:10.3389/fimmu.2025.1618883 40709171 PMC12287121

[5] Kang HM, Lee TJ, Park SE, et al. Pertussis in the post-COVID-19 era: resurgence, diagnosis, and management. Infect Chemother. 2025;57:13–30. doi:10.3947/ic.2024.0117 40183651 PMC11972920

[6] National Institute of Infectious Diseases (NIID), Japan. Pertussis in Japan, as of June 2025. Infect Agents Surveill Rep [Internet]. [cited 2025 Jul 14]. Available from: https://www.niid.go.jp/niid/en/iasr-pertussis-e.html

[7] Ministry of Health, Labour and Welfare. Number of regular immunizations [Internet]. Tokyo: Ministry of Health, Labour and Welfare; [cited 2025 Oct 1]. Available from: https://www.mhlw.go.jp/topics/bcg/other/5.html

[8] Konda T, Kamachi K, Iwaki M, et al. Distribution of pertussis antibodies among different age groups in Japan. Vaccine. 2002;20:1711–1717. doi:10.1016/S0264-410X(02)00045-2 11906757

[9] Baxter R, Bartlett J, Rowhani-Rahbar A, et al. Effectiveness of pertussis vaccines for adolescents and adults: case-control study. Br Med J. 2013;347:f4249. doi:10.1136/bmj.f4249 23873919

[10] Skoff TH, Cohn AC, Clark TA, et al. Early impact of the US Tdap vaccination program on pertussis trends. Arch Pediatr Adolesc Med. 2012;166:344–349. doi:10.1001/archpediatrics.2011.1093 22213608

[11] Kara EO, Campbell H, Ribeiro S, et al. Survey of household contacts of infants with laboratory-confirmed pertussis infection during a National Pertussis Outbreak in England and Wales. Pediatr Infect Dis J. 2017;36:140–145. doi:10.1097/INF.0000000000001378 27755466

[12] Luo F, Zheng S, Zhou Y, et al. Sources and clinical characteristics of infant pertussis in China: increasing contribution of siblings. BMC Infect Dis. 2025;25:198. doi:10.1186/s12879-025-10536-y 39930341 PMC11812223

[13] Ochi M, Nosaka N, Knaup E, et al. Recurrent apnea in an infant with pertussis due to household transmission. Clin Case Rep. 2017;5:241–245. doi:10.1002/ccr3.765 28265381 PMC5331238

[14] Leontari K, Lianou A, Tsantes AG, et al. Pertussis in early infancy: diagnostic challenges, disease burden, and public health implications amidst the 2024 resurgence, with emphasis on maternal vaccination strategies. Vaccines (Basel). 2025;13:276. doi:10.3390/vaccines13030276 40266155 PMC11945951

[15] Infectious Agent Surveillance Report (IASR). 2026;47(1):5–7. Available from: https://id-info.jihs.go.jp/surveillance/iasr/pathogens/vol47/551/551r03.html

[16] Centers for Disease Control and Prevention. Chapter 16: pertussis. In: The pink book: course textbook. 13th ed. 2024 [cited 2025 Aug]. Available from: https://www.cdc.gov/pinkbook/hcp/table-of-contents/chapter-16-pertussis.html

[17] Toubiana J, Azarnoush S, Bouchez V, et al. *Bordetella parapertussis* bacteremia: clinical expression and bacterial genomics. Open Forum Infect Dis. 2019;6:ofz122. doi:10.1093/ofid/ofz122 30976607 PMC6453521

[18] Okamoto K, Funaki T, Kidokoro S, et al. Clinical characteristics of pediatric parapertussis: a single-center case-control study. J Infect Chemother. 2025;31:102740. doi:10.1016/j.jiac.2025.102740 40436250

[19] Noble BA, Jiudice SS, Jones JD, et al. Reemergence of *Bordetella parapertussis*, United States, 2019–2023. Emerg Infect Dis. 2024;30:1058–1060. doi:10.3201/eid3005.231278 38666607 PMC11060467

[20] Asghar H, Diop OM, Weldegebriel G, et al. Environmental surveillance for polioviruses in the Global Polio Eradication Initiative. J Infect Dis. 2014;210(Suppl 1):S294–S303. doi:10.1093/infdis/jiu384 25316848 PMC10578309

[21] Medema G, Heijnen L, Elsinga G, et al. Presence of SARS-Coronavirus-2 RNA in sewage and correlation with reported COVID-19 prevalence in the early stage of the epidemic in the Netherlands. Environ Sci Technol Lett. 2020;7:511–516. doi:10.1021/acs.estlett.0c00357 37566285

[22] Wolfe MK, Duong D, Bakker KM, et al. Wastewater-based detection of two influenza outbreaks. Environ Sci Technol Lett. 2022;9:687–692. doi:10.1021/acs.estlett.2c00350

[23] Polo D, Quintela-Baluja M, Corbishley A, et al. Making waves: wastewater-based epidemiology for COVID-19 – approaches and challenges for surveillance and prediction. Water Res. 2020;186:116404. doi:10.1016/j.watres.2020.116404 32942178 PMC7480445

[24] Hovi T, Shulman LM, van der Avoort H, et al. Role of environmental poliovirus surveillance in global polio eradication and beyond. Epidemiol Infect. 2012;140:1–13. doi:10.1017/S095026881000316X 21849095

[25] Ando H, Murakami M, Kitajima M, et al. Wastewater-based estimation of temporal variation in shedding amount of influenza A virus and clinically identified cases using the PRESENS model. Environ Int. 2025;195:109218. doi:10.1016/j.envint.2024.109218 39719757

[26] Singh S, Ahmed AI, Almansoori S, et al. A narrative review of wastewater surveillance: pathogens of concern, applications, detection methods, and challenges. Front Public Health. 2024;12:1445961. doi:10.3389/fpubh.2024.1445961 39139672 PMC11319304

[27] Tiwari A, Kurittu P, Al-Mustapha AI, et al. Wastewater surveillance of antibiotic-resistant bacterial pathogens: a systematic review. Front Microbiol. 2022;13:977106. doi:10.3389/fmicb.2022.977106 36590429 PMC9798455

[28] Liu Y, Smith WJM, Gebrewold M, et al. Aircraft lavatory wastewater surveillance for movement of antimicrobial resistance genes: a proof-of-concept study. Microbiol Spectr. 2025;13. doi:10.1128/spectrum.00569-25 PMC12211000 40434126

[29] Tatti KM, Sparks KN, Boney KO, et al. Novel multitarget real-time PCR assay for rapid detection of *Bordetella* species in clinical specimens. J Clin Microbiol. 2011;49:4059–4066. doi:10.1128/JCM.00601-11 21940464 PMC3232951

[30] Kösters K, Riffelmann M, von König CHW. Evaluation of a real-time PCR assay for detection of *Bordetella pertussis* and *B. parapertussis* in clinical samples. J Med Microbiol. 2001;50:436–440. doi:10.1099/0022-1317-50-5-436 11339251

[31] Murakami M, Ando H, Yamaguchi R, et al. Evaluating survey techniques in wastewater-based epidemiology for accurate COVID-19 incidence estimation. Sci Total Environ. 2024;954:176702. doi:10.1016/j.scitotenv.2024.176702 39370003

[32] Osaka Institute of Public Health. Pertussis surveillance in Osaka Prefecture [Japanese]. Osaka Prefecture Infectious Disease Information Center; updated 2021 Jan 28. Available from: https://www.iph.pref.osaka.jp/zensu/20210128104900.html

[33] Truong C, Oudre L, Vayatis N. Selective review of offline change point detection methods. Signal Process. 2020;167:107299. doi:10.1016/j.sigpro.2019.107299

[34] Chan EMG, Kennedy LC, Wolfe MK, et al. Identifying trends in SARS-CoV-2 RNA in wastewater to infer changing COVID-19 incidence: effect of sampling frequency. PLOS Water. 2023;2:e0000088. doi:10.1371/journal.pwat.0000088

[35] Helm B, Geissler M, Mayer R, et al. Regional and temporal differences in the relation between SARS-CoV-2 biomarkers in wastewater and estimated infection prevalence: insights from long-term surveillance. Sci Total Environ. 2023;857:159358. doi:10.1016/j.scitotenv.2022.159358 36240928 PMC9554318

[36] Peccia J, Zulli A, Brackney DE, et al. Measurement of SARS-CoV-2 RNA in wastewater tracks community infection dynamics. Nat Biotechnol. 2020;38:1164–1167. doi:10.1038/s41587-020-0684-z 32948856 PMC8325066

[37] Japan Institute for Health Security. Infectious Disease Weekly Report (IDWR) [Internet]. [cited 2025 Aug 23]. Available from: https://id-info.jihs.go.jp/surveillance/idwr/index.html

[38] Gill CJ, Gunning CE, MacLeod WB, et al. Asymptomatic *Bordetella pertussis* infections in a longitudinal cohort of young African infants and their mothers. eLife. 2021;10:e65663. doi:10.7554/eLife.65663 34097599 PMC8184211

[39] Lingappa JR, Lawrence W, West-Keefe S, et al. Diagnosis of community-acquired pertussis infection: comparison of both culture and fluorescent-antibody assays with PCR detection using electrophoresis or dot blot hybridization. J Clin Microbiol. 2002;40:2908–2912. doi:10.1128/JCM.40.8.2908-2912.2002 12149350 PMC120624

[40] Kim YT, Lee K, Lee H, et al. Development of a wastewater-based infectious disease surveillance research system in South Korea. Sci Rep. 2024;14:24544. doi:10.1038/s41598-024-76614-4 39427054 PMC11490628

[41] Fu S, Du X, Xu Z, et al. The potential of wastewater monitoring as a novel surveillance tool for early warning of *Bordetella pertussis* outbreaks. Emerg Microbes Infect. 2025;14:2528537. doi:10.1080/22221751.2025.2528537 40591894 PMC12247112

[42] Edwards KM. Overview of pertussis: focus on epidemiology, sources of infection, and long term protection after infant vaccination. Pediatr Infect Dis J. 2005;24:S104–S108. doi:10.1097/01.inf.0000166154.47013.47 15931137

[43] Japan National Tourism Organization. Visitor arrivals to Japan [Internet]. Tokyo: JNTO; [cited 2025 Sep 9]. Available from: https://www.jnto.go.jp/statistics/data/visitors-statistics/2

[44] Murakami M. Bi-directional associations between mask usage and beliefs about reasons for masking before and after the downgrading of the legal status of COVID-19 in Japan: a longitudinal study. Int J Disaster Risk Reduct. 2023;97:104072. doi:10.1016/j.ijdrr.2023.104072

[45] Cole M, Simon AK, Faulkner A, et al. Comparison of *Bordetella* species identification among differing rt-PCR assays in the United States. Microbiol Spectr. 2024;12:e00783-24. doi:10.1128/spectrum.00783-24 38980022 PMC11302660

