# Supplementary material for: Early detection of pertussis related activity by wastewater surveillance of Bordetella pertussis- and Bordetella parapertussis-associated DNA concentrations in Osaka, Japan
Source: Emerg Microbes Infect. 2026 Aug 10;15(1):2717860. doi: 10.1080/22221751.2026.2717860 (PMC13495037; doi:10.1080/22221751.2026.2717860)
Supplement: Supplementary file 1 [file TEMI_A_2717860_SM8845.docx]

Supplementary Table S1. Primers and probes used for multiplex qPCR.

| **Target** | **Primer or Probe name** | **Sequence (5' - 3')** |
| --- | --- | --- |
| *IS481* | Forward primer | ATCAAGCACCGCTTTACCC |
|  | Reverse primer | TTGGGAGTTCTGGTAGGTGTG |
|  | Probe | FAM-AATGGCAAGGCCGAACGCTTCA-BHQ1 |
| *IS1001* | Forward primer | GATATCAACGGGTGACGGATC |
|  | Reverse primer | GTATGCCAACCCAGTTCGAA |
|  | Probe | FAM-TGCTGCAATCGAGCAACGTGCA-BHQ1 |
| *prn* | Forward primer | TGCCGACTGGAACAACCA |
|  | Reverse primer | GTCGGAGCCCTGGATATGG |
|  | Probe | HEX-ATCGTCAAGACCGGTGAGCGCC |

Primer and probe sequences targeting *IS481*, *IS1001*, and prn are provided in the 5’ - 3’ orientation. Probes for *IS481* and *IS1001* were labelled with 6-carboxyfluorescein (FAM) at the 5’ end and Black Hole Quencher 1 (BHQ1) at the 3’ end, whereas the *prn* probe was labelled with hexachloro-fluorescein (HEX) at the 5’ end.

qPCR, Quantitative PCR

Supplementary Table S2. Limit of detection of *IS481* and *prn* by real-time PCR

|  | **DNA copies** | **Ct value** | |
| --- | --- | --- | --- |
|  |  | ***IS481*** | ***prn*** |
| gDNA | 10^6^ | 15.9 | 27.7 |
|  | 10^5^ | 18.8 | 30.9 |
|  | 10^4^ | 22.1 | 34.2 |
|  | 10^3^ | 25.4 | 37.7 |
|  | 10^2^ | 28.5 | 41.3 |
|  | 10^1^ | 32.0 | N.D. |
|  | 10^0^ | 36.2 | N.D. |
| pre-amplified DNA | 10^6^ | 7.1 | 20.4 |
|  | 10^5^ | 9.0 | 24.0 |
|  | 10^4^ | 12.3 | 26.0 |
|  | 10^3^ | 15.4 | 29.3 |
|  | 10^2^ | 17.4 | 32.7 |
|  | 10^1^ | 21.7 | N.D. |
|  | 10^0^ | 22.2 | N.D. |

Serial dilutions of *B. pertussis* genomic DNA (gDNA) were subjected to preamplification using the *IS481* and *prn* primers listed in Table S1. Preamplification was performed with KOD Plus Ver.2 (KOD-211; TOYOBO, Osaka, Japan) for 20 cycles, in accordance with the manufacturer’s instructions. After purification of amplified DNA by AMPure XP, both the original gDNA and the preamplified products (preAMP) were subsequently analysed by real-time PCR using the corresponding primers and probes shown in Table S1. Real-time PCR was conducted with One Step PrimeScript™ III RT-qPCR Mix with UNG (Takara Bio, Shiga, Japan), following the manufacturer’s protocol.

Supplementary Table S3. Sensitivity analysis of threshold-based onset detection using alternative threshold values and consecutive-week requirements

| **Threshold** | **Consecutive weeks** | **Wastewater onset week** | **Case onset week** | **Onset difference weeks  (cases - wastewater)** |
| --- | --- | --- | --- | --- |
| Mean + 1.5 SD | 4 | Apri 8-14, 2024  (2024 week 15) | July 22-28, 2024  (2024 week 30) | 15 |
| Mean + 2.0 SD^a^ | 4 | Apri 8-14, 2024  (2024 week 15) | July 22-28, 2024  (2024 week 30) | 15 |
| Mean + 2.5 SD | 4 | Apri 1-7, 2024  (2024 week 14) | July 22-28, 2024  (2024 week 30) | 16 |
| Mean + 2.0 SD | 2 | Apri 8-14, 2024  (2024 week 15) | July 22-28, 2024  (2024 week 30) | 15 |
| Mean + 2.0 SD | 3 | Apri 8-14, 2024  (2024 week 15) | July 22-28, 2024  (2024 week 30) | 15 |
| Mean + 2.0 SD | 5 | June 24-30, 2024  (2024 week 26) | July 22-28, 2024  (2024 week 30) | 4 |

Thresholds were calculated separately for each time series using the baseline mean + 1.5 SD, +2.0 SD, or +2.5 SD. Weekly reported case counts from January 2022 through December 2023 were used as the case baseline, and wastewater *IS481* DNA concentrations from October through December 2023 were used as the wastewater baseline. Onset was defined as the first week of the specified number of consecutive weeks above the threshold. The onset difference was calculated as the case onset week minus the wastewater onset week; positive values indicate that the increase in wastewater *IS481* DNA concentration preceded the increase in reported cases.
^a^ Primary analytical specification.
